# Association of anticipated HIV testing stigma and provider mistrust on preference for HIV self-testing among cisgender men who have sex with men in the Philippines

**DOI:** 10.1186/s12889-022-14834-x

**Published:** 2022-12-16

**Authors:** Olivia T. Sison, Emmanuel S. Baja, Amiel Nazer C. Bermudez, Ma. Irene N. Quilantang, Godofreda V. Dalmacion, Ernest Genesis Guevara, Rhoda Myra Garces-Bacsal, Charlotte Hemingway, Miriam Taegtmeyer, Don Operario, Katie B. Biello

**Affiliations:** 1 Department of Epidemiology, Brown University School of Public Health, Providence, RI USA; 2 The Philippine Health Initiative for Research, Service, and Training, Brown University Global Health Initiative, Providence, RI USA; 3 Institute of Clinical Epidemiology, National Institutes of Health, University of the Philippines Manila, Manila, Metro Manila, Philippines; 4 Department of Clinical Epidemiology, College of Medicine, University of the Philippines Manila, Manila, Metro Manila, Philippines; 5 Department of Epidemiology and Biostatistics, College of Public Health, University of the Philippines Manila, Manila, Metro Manila, Philippines; 6 Department of Behavioral and Social Sciences, Brown University School of Public Health, Providence, RI USA; 7 Department of Behavioral Sciences, College of Arts and Sciences, University of the Philippines Manila, Manila, Metro Manila, Philippines; 8 Department of Special Education, College of Education, United Arab Emirates University, P.O. Box 15551, Al Ain, UAE; 9 Department of International Public Health, Liverpool School of Tropical Medicine, Pembroke Palace Liverpool, Liverpool, L3 5QA UK; 10 Tropical Infectious Disease Unit, Royal Liverpool University Hospital, Prescot Street, Liverpool, L7 8XP UK; 11 Department of Behavioral, Social, and Health Education Sciences, Rollins School of Public Health, Emory University, Atlanta, GA USA

**Keywords:** HIV self-testing, Men who have sex with men (MSM), Anticipated HIV testing stigma, Provider mistrust, Philippines

## Abstract

**Background:**

New HIV infections in the Philippines are increasing at an alarming rate. However, over three quarters of men who have sex with men (MSM) have never been tested for HIV. HIV self-testing (HIVST) may increase overall testing rates by removing barriers, particularly fear of stigmatization and mistrust of providers. This study aimed to determine if these factors are associated with preference for HIVST among Filipino cisgender MSM (cis-MSM), and whether there is an interaction between anticipated HIV testing stigma and provider mistrust on preference for HIVST.

**Methods:**

We conducted secondary analysis of a one-time survey of 803 cis-MSM who were recruited using purposive sampling from online MSM dating sites and MSM-themed bar locations in Metro Manila, Philippines. Summary statistics were computed to describe participant characteristics. Multivariable modified Poisson regression analyses were conducted to determine if anticipated HIV testing stigma and provider mistrust were associated with preference for HIVST among cis-MSM. Other variables such as age, education, monthly income, relationship status, HIV serostatus, and knowing where to get HIV testing were the minimal sufficient adjustment set in the analyses.

**Results:**

Average age of participants was 28.6 years (SD = 8.0); most had received college degrees (73%) and were employed (80%). Most respondents (81%) preferred facility-based testing, while 19% preferred HIVST. A high percentage of participants reported anticipated HIV testing stigma (66%) and provider mistrust (44%). Anticipated HIV testing stigma (aPR = 1.51; 95% CI = 1.01–2.25, *p* = 0.046) and provider mistrust (aPR = 1.49; 95% CI = 1.07–2.09, *p* = 0.020) were independently associated with a preference for HIVST. There was a positive, additive interaction between provider mistrust and anticipated HIV testing stigma on preference for HIVST (RERI = 1.13, 95% CI: 0.20–2.06; *p* = 0.017), indicating that the association between anticipated HIV testing stigma and preference for HIVST is greater among those with provider mistrust compared to those without provider mistrust.

**Conclusions:**

HIVST should be offered as a supplement to traditional facility-based HIV testing services in the Philippines to expand testing and reach individuals who may not undergo testing due to anticipated HIV testing stigma and provider mistrust.

**Supplementary Information:**

The online version contains supplementary material available at 10.1186/s12889-022-14834-x.

## Background

The rate of increase in new HIV infections in the Philippines is alarming [1]. On average, 42 new HIV cases per day were diagnosed in 2022 compared to 25 cases per day in 2016 and nine cases per day in 2012 [2–4]. Eighty-five percent of all diagnosed HIV cases in the Philippines from 2017 to 2022 were among men who have sex with men (MSM), the majority of whom were adolescents (30%) and young adults (50%) [3].

The HIV prevention continuum highlights the importance of HIV testing as an essential first step in both prevention and treatment cascades [5]. However, studies in Europe, the United States (US), South Africa, and the Philippines reported that low HIV testing uptake is associated with: sociodemographic factors such as younger age, lower education level, and higher socioeconomic status; lack of accessibility to services; lack of awareness of HIV testing and counseling; number of sexual partners; health care provider factors (e.g. onward referral due to avoidance of the issue of HIV testing); unfriendly testing environments; and psychosocial factors such as fear of rejection and disclosure, and HIV-related stigma and discrimination [6–11].

Voluntary facility-based testing is the primary model of HIV testing in the Philippines [12–14]. The most common facilities providing HIV testing services in the Philippines include hospitals, health clinics, or community-based organizations [12–14]. According to the Philippine Department of Health (DOH) Integrated HIV Behavioral and Serologic Surveillance data, HIV testing uptake among key populations (e.g., sex workers, MSM, people who inject drugs, transgender people) in the Philippines is low. Only 22 to 28% of MSM in the Philippines have received HIV testing between 2015 to 2019 [15, 16]. During the first year of the COVID-19 pandemic, HIV testing in the Philippines decreased by 61% due to community restrictions that disrupted access to facility-based HIV testing services [14, 17].

To achieve the United Nations 90–90-90 global HIV targets, with the goal of diagnosing 90% of all people living with HIV (PLHIV), providing antiretroviral therapy (ART) to 90% of those diagnosed with HIV, and achieving viral suppression for 90% of those receiving ART by 2020, the World Health Organization (WHO) launched a set of consolidated guidelines in 2016 for HIV testing services [5]. The guidelines emphasize the promise of HIV self-testing (HIVST) as an additional approach to increase HIV testing coverage, especially among MSM and other key populations [18]. Given the significant progress in addressing HIV globally, the United Nations updated the global HIV targets in 2020 and increased them to 95–95-95 [19]. This reflects the intention to diagnose 95% of all PLHIV by 2025. In response, the Philippine DOH issued an Administrative Order (AO No. 2022–0035) in August 2022 to include HIVST as one of the HIV testing options available at the primary care level in the country [20].

Previous studies in Australia, the US, Africa, and Hong Kong have shown that HIVST was generally acceptable among MSM, and that it increased HIV testing coverage because of its convenience while ensuring confidentiality and privacy [21, 22]. Convenience, privacy, and confidentiality are motivating factors for HIVST in the Philippines [23]. A qualitative study in 2017 of key informants and stakeholders from the MSM and transgender women (TGW) communities in the Philippines found HIVST was acceptable as an additional approach to HIV testing services [12]. Due to limited access to facility-based testing services during the COVID-19 pandemic, demonstration studies were conducted in Metro Manila and Western Visayas in the Philippines and showed that HIVST was acceptable and feasible among MSM and TGW, and reactivity rate was 8–10% [23–25]. In these demonstration studies, HIVST was made available using courier delivery methods and via in-clinic appointments.

The acceptability and feasibility from these demonstration studies showed the promise of HIVST as a strategy to increase HIV testing coverage among key populations in the Philippines. However, factors contributing to HIVST uptake in the country must be further studied as there still remain some concerns regarding accessing the service, particularly the lack of privacy and maintenance of confidentiality during delivery of HIVST kits [23]. Studies on preference for HIVST, including identifying motivating factors as well as barriers to use, can guide HIVST roll out in the country.

Studies in the US found that experiencing stigma and medical-related mistrust have each been associated with lower engagement in care or underutilization of health services [11, 26–30]. In particular, anticipated stigma was found to be a significant predictor of HIV testing behavior [31, 32]. Anticipated stigma refers to an individual’s expectation to experience prejudice and discrimination from others in the future [33]. In a scoping review of health-related stigma outcomes in low- and middle-income countries, anticipated stigma was associated with decreased voluntary HIV testing [34]. Our study explored two specific areas of medical-related mistrust: mistrust in health care providers and mistrust in the health care facility [35]. Higher levels of provider mistrust among people living with HIV have previously been associated with suboptimal engagement with health care [11]. Provider mistrust and stigma are important determinants for poorer health outcomes because these potentially modifiable factors might influence health care utilization and thus affect the overall health among the high-risk groups. The additive effects of experiencing both anticipated stigma and provider mistrust have received limited research attention and deserve attention. A systematic review of research conducted in multiple global contexts found that HIVST is particularly promising among MSM who often encounter structural barriers, such as stigma and discrimination, that deter them from accessing HIV-related services [36, 37].

To date, there is a paucity of data in the Philippines on preferences for HIVST among MSM and correlates of HIVST preferences in this population. This study aimed to (i) determine the percentage of cis-MSM in the Philippines who prefer HIVST rather than the traditional facility-based HIV testing services, (ii) determine if anticipated HIV testing stigma and provider mistrust were associated with preference for HIVST among Filipino cis-MSM, and (iii) examine whether there is an interaction between anticipated HIV testing stigma and provider mistrust on preference for HIVST.

## Methods

### Study design and setting

This study analyzed data from the HIV Gaming, Engaging, and Testing (HIV GET) Project, which had an overarching aim to develop and evaluate a mobile game application to address identified barriers to HIV services [38]. Targeted messaging was used to recruit HIV GET study participants via posting of study flyers and in-person outreach at venues where MSM frequent, and advertisements on MSM dating sites (e.g., Grindr, Planet Romeo, and GROWLr) and MSM-themed bar locations in Quezon City, Philippines. Participants were eligible if they were at least 18 years old, assigned male sex at birth, self-identified as MSM, and were able to give informed consent. Given the overarching project aims, HIV status was not a criterion for enrollment. Using purposive sampling, a total of 899 participants completed the survey between October and November 2016. We excluded in the analytic sample participants who did not identify as cis-MSM and those who self-reported to be HIV positive. A total of 803 cis-MSM was included in this secondary analysis. More than a quarter of these participants resided outside Metro Manila.

### Procedures

Screening questions were used to identify eligible participants, and those who were eligible were redirected to the main survey questionnaire page. Participants recruited from bar locations completed a survey administered via mobile tablet. Participants recruited via social media platforms responded to study informational messages posted on targeted websites. Those who clicked the advertisement on these sites were redirected to the informed consent page for the online survey. Survey questions were in English and Tagalog (local language). The survey questions were developed based on findings from an unpublished qualitative study among MSM, TGW, and HIV service providers [38]. Survey participants were not compensated in this study.

### Measures

#### Dependent variable

*Preferred HIV testing method.* Participants selected their preferred HIV testing method from the following options: (1) hospital-based testing, (2) clinic-based testing *(social hygiene clinics)*, (3) home-based testing with a health worker, (4) community-based testing with a health worker, and (5) self-testing. This was coded as a binary variable (HIV self-testing vs. any other preferred option).

#### Independent variables

We assessed *anticipated HIV testing stigma and provider mistrust* as exposures of interest*.* As noted, items for both constructs were based on preliminary findings from a qualitative study of HIV testing preferences among key populations in the Philippines [38]. Anticipated HIV testing stigma was measured based on respondents’ level of agreement with the following statements: (1) *I feel like I would be stigmatized going to an HIV/AIDS testing facility,* (2) *I worry about being recognized at the HIV/AIDS testing facility,* (3) *I feel like the staff would disrespect me* (Cronbach *α* = 0.80)*.* Provider mistrust was assessed based on respondents’ level of agreement with the following statements: (1) *I don’t think there will be anyone in the HIV/AIDS testing facility that I can trust to talk to*, (2) *I don’t trust the counselors at the HIV/AIDS testing facility*, (3) *I don’t trust the people that take your blood at the HIV/AIDS testing facility*, (4) *I don’t trust the results you get at the HIV/AIDS testing facility* (Cronbach *α* = 0.89). The level of agreement for the statements was measured using a 7-point Likert scale (strongly disagree to strongly agree), and responses were dichotomized. If respondents agreed or strongly agreed to at least one of the statements indicative of anticipated HIV testing stigma and provider mistrust, they were coded as experiencing anticipated HIV testing stigma and provider mistrust, respectively.

#### Sociodemographic and other participant characteristics

The respondent’s age in years was categorized as 18–24, 25–34, ≥35. Educational attainment was coded as a binary variable (graduated from college or higher vs. some college and below). Monthly income was categorized based on a defined poverty threshold as 10,000 pesos and below (≤USD 207) or more than 10,000 pesos (>USD 207) [39]. Participants’ relationship status was classified as follows: single, not looking for a relationship; single, looking only for serious relationship; single, looking only for casual relationships; in a relationship, exclusive; and in a relationship, open. Participants’ recent HIV testing experience was probed (never been tested, past 12 months, more than a year ago), and their self- reported awareness of HIV status was categorized as HIV negative, HIV positive, unsure, did not want to answer. Those who self-reported to be HIV positive were excluded in the analytic sample. They were also asked if they knew where to get HIV testing (yes vs. no). Survey respondents were asked about their sexual orientation with the following response options: (1) heterosexual, (2) gay/homosexual, (3) bisexual, (4) discreet (do not openly disclose sexual activities), (5) not in any category.

### Data analysis

Frequencies and percentages were calculated for categorical variables. Means, standard deviations, and ranges were calculated for continuous variables. To determine the internal consistency of our scale variables, we computed for Cronbach’s alpha. Separate bivariable modified Poisson regressions were performed to estimate the prevalence ratios for the association between preference for HIVST and the following covariates: age, relationship status, level of education, employment status, monthly income, knowing where to get HIV testing, recent HIV test, awareness of HIV status, anticipated HIV testing stigma, and provider mistrust. Modified Poisson regression was used to estimate prevalence ratios rather than odds ratios because the dependent variable was not rare [40, 41]. Directed acyclic graphs (DAG) were constructed to determine the minimum set of covariates to adjust for in the analysis of the association between anticipated HIV testing stigma and preference for HIVST, and provider mistrust and preference for HIVST using Causal Fusion (See Additional file 1) [42]. All variables included in the DAG, as well as their interrelationships were determined a priori through expert knowledge and literature review. Using the DAGs constructed for this study, the association of the exposure variables with preference for HIVST was considered unbiased given a set of covariates ***S*** if, after conditioning on ***S***, the open paths between the exposure variables and preference for HIVST were exactly the directed paths from the exposure variables to preference for HIVST. A variable was considered a component of ***S*** if conditioning on it blocks biasing backdoor paths [43]. For our study, the minimal sufficient adjustment set includes age [8, 31, 44–46], level of education [8, 45–47], monthly income [10, 46], relationship status [8, 48], awareness of HIV status [6, 49], and knowing where to get HIV testing [7–10]. Multivariable modified Poisson regression analyses were performed to estimate prevalence ratios for the association of exposure variables and preference for HIVST, adjusting for the covariates described previously. Separate generalized linear models with Poisson distribution and log link were constructed with anticipated HIV testing stigma and provider mistrust as independent variables in the two models (Models 1 and 2). A third model was constructed to explore the interaction between anticipated HIV testing stigma and provider mistrust on preference for HIVST (Model 3). The statistical interaction on the additive scale between anticipated HIV testing stigma and provider mistrust was determined by estimating the relative excess risk due to interaction (RERI) and its 95% confidence interval (CI), following the method outlined by VanderWeele [50]. A RERI of greater than zero denotes a positive interaction on the additive scale between anticipated HIV testing stigma and provider mistrust on preference for HIVST. Positive interaction in this study would denote a stronger association between anticipated HIV testing stigma and preference for HIVST among those who have provider mistrust compared to those who do not have provider mistrust. Estimating interaction on the additive scale is considered more relevant in evaluating the public health impact, as it suggests which exposure group to target for an intervention [50]. The ratio of prevalence ratios was also reported as the measure of multiplicative interaction, where a ratio of one means no interaction and a ratio bigger than one indicates positive interaction on the multiplicative scale. Adjusted prevalence ratios (aPR) and their 95% CIs relating the independent variables and preference for HIVST are presented [51]. Data management and statistical analyses were performed using Stata version 16 [52].

## Results

Table 1 summarizes the overall sample characteristics of the participants. In brief, survey participants were between 18 and 61 years old with an average age of 28.6 years (SD = 8.0), mostly with college degrees (73%) and employed (80%). The majority of the sample identified as gay/homosexual, more than a quarter identified as bisexual, while 17% did not openly disclose their sexual orientation. More than half (57%) reported to be HIV negative and one-third were unsure of their HIV status. Almost half (45%) of the respondents had undergone HIV testing in the past 12 months. However, 37% (*N* = 293) had never been tested for HIV, although 70% of all participants knew where to get HIV testing. Almost two out of ten (19%) preferred HIV self-testing over in-person, facility-based HIV testing methods.

**Table 1:** Profile of Filipino cis-MSM survey participants (*n* = 803)^a^

	Frequency	%
Age (years)		
Mean (SD^b^)	28.6 (8.0)	
Range	18–61	
18–24	278	34.6
25–34	368	45.8
> 35	150	18.7
Sexual orientation		
Heterosexual	33	4.1
Gay/homosexual	393	48.9
Bisexual	228	28.4
Do not openly disclose	137	17.1
Do not place self in any sexual category	12	1.5
Relationship status		
Single, not looking for a relationship	161	20.0
Single, looking only for serious relationship	295	36.7
Single, looking only for casual relationship	103	12.8
In a relationship, exclusive	159	19.8
In a relationship, open	85	10.6
Education		
College undergraduate & below	185	23.0
At least college graduate	585	72.9
Employment status		
Unemployed	105	13.1
Employed	641	79.8
Do not want to disclose	57	7.1
Monthly income (Philippine peso)		
10,000 & below	152	18.9
Above 10,000	519	64.6
Recent HIV test		
Past 12 months	363	45.2
Never been tested	293	36.5
More than a year ago	147	18.3
Awareness of their HIV status		
HIV negative	461	57.4
Unsure	287	35.8
Do not want to disclose	55	6.9
Know where to get tested		
Yes	560	69.7
No	243	30.3
Preferred HIV testing method		
Hospital/Clinic/Home or Community-based with a health care worker	648	80.7
Self-testing	155	19.3

^a^ Percentages may not add up to 100% due to missing data

^b^SD- standard deviation

Table 2 presents participants’ level of agreement with items assessing for anticipated HIV testing stigma and provider mistrust. Overall, 66% (*N* = 519) agreed to at least one statement pertaining to anticipated HIV testing stigma, while 44% (*N* = 338) reported mistrust of health care providers in the testing facilities.

**Table 2:** Level of agreement on anticipated HIV testing stigma and provider mistrust among Filipino cis-MSM

	Frequency	%
**Anticipated HIV testing stigma**^a^	**519**	**66.3**
I feel like I would be stigmatized going to an HIV/AIDS testing facility	421	53.8
I worry about being recognized at the HIV/AIDS testing facility	417	53.3
I feel like the staff would disrespect me	249	31.8
**Provider mistrust**^b^	**338**	**43.8**
I don’t think there will be anyone in the HIV/AIDS testing facility that I can trust to talk to	261	33.9
I don’t trust the counselors at the HIV/AIDS testing facility	188	24.4
I don’t trust the people that take your blood at the HIV/AIDS testing facility	189	24.5
I don’t trust the results you get at the HIV/AIDS testing facility	148	19.2

^a^If the participant agreed to at least one question on anticipated HIV testing stigma questions, the person was considered with anticipated stigma (*n* = 783)

^b^If the participant agreed to at least one question on provider mistrust questions, the person was considered with provider mistrust (*n* = 771)

Table 3 presents (i) bivariable associations between all covariates and preference for HIVST, and (ii) adjusted associations of anticipated HIV testing stigma and provider mistrust with preference for HIVST. Participants who preferred HIVST tended to be older, unsure of their HIV status, did not know where to get HIV testing, and had never been tested for HIV nor was currently engaged in routine testing. Both anticipated HIV testing stigma and provider mistrust were associated with participants’ preference for HIVST over the other HIV testing methods. In adjusted analyses, anticipated HIV testing stigma was associated with a 51% increase in the prevalence of HIVST preference (aPR = 1.51; 95% CI = 1.01–2.25, *p* = 0.046), and provider mistrust was associated with a 49% increase in the prevalence of HIVST preference (aPR = 1.49; 95% CI = 1.07–2.09, *p* = 0.020).

**Table 3:** Crude and adjusted associations between covariates and preference for HIVST among Filipino cis-MSM^a^

Characteristic	Total *n*	HIVST		Facility/community-based testing		Crude PR (95% CI)	Adjusted PR (95% CI)
		*n* 167	% 18.6	*n* 732	% 81.4		
Age							
18–24	278	34	12.2	244	87.8	1.0	
25–34	368	82	22.3	286	77.7	1.82 (1.26–2.63)^*^	
> 35	150	39	26.0	111	74.0	2.13 (1.40–3.22)^**^	
Relationship status							
Single, not looking for a relationship	161	21	13.0	140	87.0	1.0	
Single, looking only for serious relationship	295	66	22.4	229	77.6	1.72 (1.09–2.70)^*^	
Single, looking only for casual relationship	103	30	29.1	73	70.9	2.23 (1.35–3.68)^*^	
In a relationship, exclusive	159	20	12.6	139	87.4	0.96 (0.54 – 1.71)	
In a relationship, open	85	18	21.2	67	78.8	1.62 (0.92–2.88)^+^	
Education							
College undergraduate & below	185	28	15.1	157	84.9	1.0	
At least college graduate	585	116	19.8	469	80.2	1.31 (0.90–1.91)	
Employment status^a^							
Unemployed	105	15	14.3	90	85.7	1.0	
Employed	641	128	20.0	513	80.0	1.40 (0.85–2.29)	
Monthly income (Philippine peso)							
10 K & below	152	24	15.8	128	84.2	1.0	
Above 10 K	519	105	20.2	414	79.8	1.28 (0.85–1.92)	
Know where to get tested							
Yes	560	88	15.7	472	84.3	1.0	
No	243	67	27.6	176	72.4	1.75 (1.33–2.32)^**^	
Recent HIV test							
Past 12 months	363	35	9.6	328	90.4	1.0	
Never tested	293	82	28.0	211	72.0	2.90 (2.02–4.18)^**^	
> 1 year ago	147	38	25.9	109	74.2	2.68 (1.77–4.07)^**^	
Awareness of their HIV status							
Negative	461	64	13.9	397	86.1	1.0	
Unsure	287	79	27.5	208	72.5	1.98 (1.48–2.66)^**^	
Anticipated HIV testing stigma							
No	264	38	14.4	226	85.6	1.0	1.0
Yes	519	114	22.0	405	78.0	1.53 (1.09–2.14)^*^	1.51 (1.01–2.25)*
Provider mistrust							
No	433	65	15.0	368	85.0	1.0	1.0
Yes	338	81	24.0	257	76.0	1.60 (1.19–2.14)^*^	1.49 (1.07–2.09)*

^a^Numbers may not sum to the total sample size due to missing data

Adjusted analyses included *N* = 595 for stigma and *N* = 586 for provider mistrust due to missing data for some of the covariates.

Adjusted PRs are adjusted for age, education, HIV status, know where to get tested, monthly income, relationship status.

CI: confidence interval; PR: Prevalence Ratio; HIVST: HIV self-testing.

^+^*p* < 0.10; *p < 0.05; **p < 0.001

There was a significant positive, additive interaction between provider mistrust and anticipated HIV testing stigma on preference for HIVST (RERI = 1.13, 95% CI: 0.20–2.06; *p* = 0.017), indicating the association between anticipated HIV testing stigma and preference for HIVST is greater among those with provider mistrust compared to those without provider mistrust. On the multiplicative scale, there was a positive interaction trend between provider mistrust and anticipated HIV testing stigma on preference for HIVST, but this fell short of statistical significance (*p = 0.168*). Table 4 summarizes the stratified results. After adjusting for all covariates, provider mistrust was positively and significantly associated with preference for HIVST (aPR = 1.54; 95% CI = 1.00–2.36, *p* = 0.050) among those with anticipated HIV testing stigma, and anticipated HIV testing stigma was also positively but non-significantly associated with preference for HIVST among respondents who reported to have provider mistrust (aPR = 3.71; 95% CI = 0.67–20.39, *p* = 0.132).

**Table 4:** Interaction between anticipated HIV testing stigma and provider mistrust on preference for HIV self-testing among Filipino cis-MSM^a^

Provider mistrust	Anticipated stigma				PR (95% CI) for anticipated stigma within strata of provider mistrust
	No		Yes		
	*n* HIVST/ other testing	PR (95% CI)	*n* HIVST/ other testing	PR (95% CI)	
No	35/200	1.00	30/166	1.06 (0.63–1.79); *p = 0.823*	1.06 (0.63–1.79); *p = 0.823*
Yes	3/26	0.44 (0.08–2.44); *p = 0.347*	76/226	1.63 (1.06–2.50); *p = 0.025*	3.71 (0.67–20.39); *p = 0.132*
PR (95% CI) for provider mistrust within strata of anticipated stigma		0.44 (0.08–2.44); *p = 0.347*		1.54 (1.00–2.36); *p = 0.050*	

Measure of interaction on additive scale: RERI (95% CI) = 1.13 (0.20–2.06); ***p*** = 0.017.

Measure of interaction on multiplicative scale: ratio of PRs (95% CI) = 3.49 (0.59–20.71); ***p*** = 0.168.

PRs are adjusted for age, education, HIV status, know where to get tested, monthly income, relationship status.

CI: confidence interval; PR: Prevalence Ratio; RERI: relative excess risk due to interaction.

^a^Adjusted analyses included *N* = 580 due to missing data for some of the covariates

## Discussion

Our study is the first known quantitative assessment of preference for HIVST among cis-MSM in the Philippines, who comprise more than 80% of all diagnosed HIV cases in the country [4]. Over 60% of survey participants had ever been tested for HIV with 45% having been tested in the past 12 months. This percentage was higher than the reported estimate that only 22% of Filipino MSM had ever been tested for HIV [15]. It is possible that because survey respondents were recruited in social venues where HIV prevention and testing messages exist, members of this study sample were more likely to have been tested for HIV.

Almost one fifth of the survey participants (155/803) preferred HIVST. This subgroup tended to be older, unsure of their HIV status, did not know where to get HIV testing, and had never been tested for HIV nor was currently engaged in routine testing. Compared to previous studies in other parts of the world which found that HIVST was highly acceptable to target users including MSM [12, 44, 53–56], the percentage of MSM in our sample who preferred HIVST was lower than expected. One possible reason for the lower preference for HIVST in this group is the moderate level of awareness about HIVST in the study sample. At the time of this report, only 56% of MSM in the Philippines had heard about HIVST [57]. Moreover, at the time of data collection for this study, HIVST was also not yet included in the national HIV testing policies and guidelines in the Philippines and WHO-approved self-test kits are unavailable. These may have contributed to lower than expected levels of preference for HIVST observed here.

More than half of the participants in the sample preferred facility-based HIV testing. Similar studies in the United Kingdom (UK) and Ireland conducted after the timing of our survey also found MSM still prefer facility-based HIV testing [58, 59]. Some possible reasons for choosing facility-based HIV testing include the opportunity for engagement with a live, in-person counselor and access to ancillary services (e.g., referrals to mental health or social service programs; linkage to HIV care for those testing HIV positive) provided in the testing facility [21]. Preference for HIVST in this population is likely to increase as awareness of and trust in this testing modality grow in the Philippines. This was evident during the COVID-19 pandemic when community quarantines were enforced and access to in-clinic testing were limited [17, 25]. HIV-focused community-based organizations (CBO) reported that delivery of HIV-related services, including HIVST, and conduct of HIVST with assistance by an HIV counselor via online platforms ensured that HIV testing services were continuously accessible to key populations and even first time users during the pandemic [17, 25, 60]. Given the confidentiality, privacy, and independence that HIVST provides, as well as the convenience of accessing it, if accessed via courier services facilitated by CBOs or HIV treatment facilities, HIVST proves to be a promising HIV-related service in the Philippines [17, 23, 25, 60]. Thus, HIVST can be considered as supplementary to facility-based testing rather than needing to replace traditional HIV testing services [61]. Traditional HIV testing and HIVST may co-exist in the HIV testing services framework [62]. In fact, a meta-analysis conducted in 2017 showed that providing HIVST in addition to traditional testing modality significantly increased HIV testing uptake [22, 63].

Provider mistrust and stigma are salient barriers to healthcare utilization and affect the overall health among vulnerable high-risk groups such as MSM [11, 26–28, 30–32, 64]. A global systematic review including 18 studies reported that preferences for HIVST was due to increased convenience and confidentiality, especially among stigmatized populations and decreased test-associated stigma [65]. A significant percentage of our study participants reported anticipated HIV testing stigma and mistrust of health care providers in the testing facilities. Both anticipated HIV testing stigma and provider mistrust were associated with preference for HIVST. These findings are consistent with previous studies reporting that stigma and physician mistrust were associated with HIV testing behavior and utilization of health services among MSM [11, 27, 28, 30–32]. Our findings suggest an opportunity to increase HIV testing in the Philippines by offering HIVST as an option for individuals concerned about stigma and provider mistrust. Moreover, efforts to rebuild trust in health care providers and address sources of stigma among Filipino MSM and other key populations are also needed to improve HIV testing uptake and engage members of these groups in necessary healthcare services. Indeed, as an early effort to address this concern, a “sundown clinic” (i.e., one that operates beyond traditional “daylight” working hours) was established in Quezon City, Philippines in 2012 and was considered non-stigmatizing and a safe space for MSM and transgender people for receiving HIV testing and counseling services [66]. However, future efforts to scale-up this initiative to different areas in the country are much needed.

There are several limitations of the study. First, participants were recruited via MSM social venues and mobile dating apps, and most of the participants were between 18 to 34 years old and were highly educated and employed. Thus, this study sample population may not reflect the Philippines’ general MSM population. Second, sexual behaviors were not measured, and HIV status was assessed via self-report. Therefore, the risk for HIV infection/transmission in our sample population is unclear. Third, social desirability may have led to overreporting of recent HIV testing and underreporting of HIV serostatus. Fourth, residual confounding may have been introduced due to possible unmeasured confounders, such as health service delivery and health provider factors that were not assessed in our study. We recommend future research studies investigate these multilevel determinants, and possibly examine contextual effects. Future research studies should look into other forms of stigma, the differences in experiences in health care stigma, and the difference in attitudes towards HIVST based on gender identity and sexuality. Fifth, exposure misclassification may have been introduced due to the limitations inherent to the secondary analysis of an existing dataset; however, we assume that these exposure misclassifications are likely to be non-differential with respect to the outcome, given that exposure definitions were developed post-data collection stage. A total of 223 (28%) of the observations were excluded in the adjusted analysis due to missing information mostly on income and some other covariates. There was no evidence of an association between missingness and our primary outcome, and that the complete case analysis estimates of exposure associations can be asymptotically unbiased [67]. Finally, due to the cross-sectional nature of the study, findings are descriptive and preclude temporal or causal inferences.

## Conclusions

This study conducted among cis-MSM in the Philippines suggests that one out of five cis-MSM preferred HIVST over the traditional HIV testing strategies. An upsurge in preference for HIVST among cis-MSM in the Philippines may increase with expanded campaigns to raise awareness, understanding, and access to HIVST methods in the future. Moreover, Philippines national HIVST guidelines and access to WHO-approved HIVST materials are likely to increase levels of awareness, acceptability, and uptake of HIVST. Therefore, our findings reported here offer a baseline description of preference for HIVST prior to the implementation of structural programs to promote HIVST. In addition, anticipated HIV testing stigma and mistrust of health care providers in the testing facilities were reported, and both factors were associated with a higher prevalence of HIVST preference. HIVST represents a compelling complementary option to traditional HIV testing services in the Philippines, which can expand testing among cis-MSM who do not undergo testing due to anticipated HIV testing stigma and provider mistrust and provide differentiated HIV testing service delivery options to specific subgroups among key populations.

## Supplementary Information

**Additional file 1.**

## Data Availability

The datasets generated and/or analyzed during the current study are available in the Harvard Dataverse repository, 10.7910/DVN/PFUMZM.

## Abbreviations

AIDS: Acquired immunodeficiency syndrome
CI: Confidence Interval
DAG: Directed acyclic graph
DOH: Department of Health
HIV: Human immunodeficiency virus
HIV GET: HIV Gaming, Engaging, and Testing
HIVST: HIV self-testing
MSM: Men who have sex with men
PLHIV: People Living with HIV
PR: Prevalence Ratio
RERI: Relative Excess Risk due to Interaction
SD: Standard Deviation
TGW: Transgender women
WHO: World Health Organization
