# Supplementary material for: Association of anticipated HIV testing stigma and provider mistrust on preference for HIV self-testing among cisgender men who have sex with men in the Philippines
Source: BMC Public Health. 2022 Dec 16;22:2362. doi: 10.1186/s12889-022-14834-x (PMC9756449; doi:10.1186/s12889-022-14834-x)
Supplement: Supplementary file 1 — Additional file 1. [file 12889_2022_14834_MOESM1_ESM.pptx]

## Slide 1
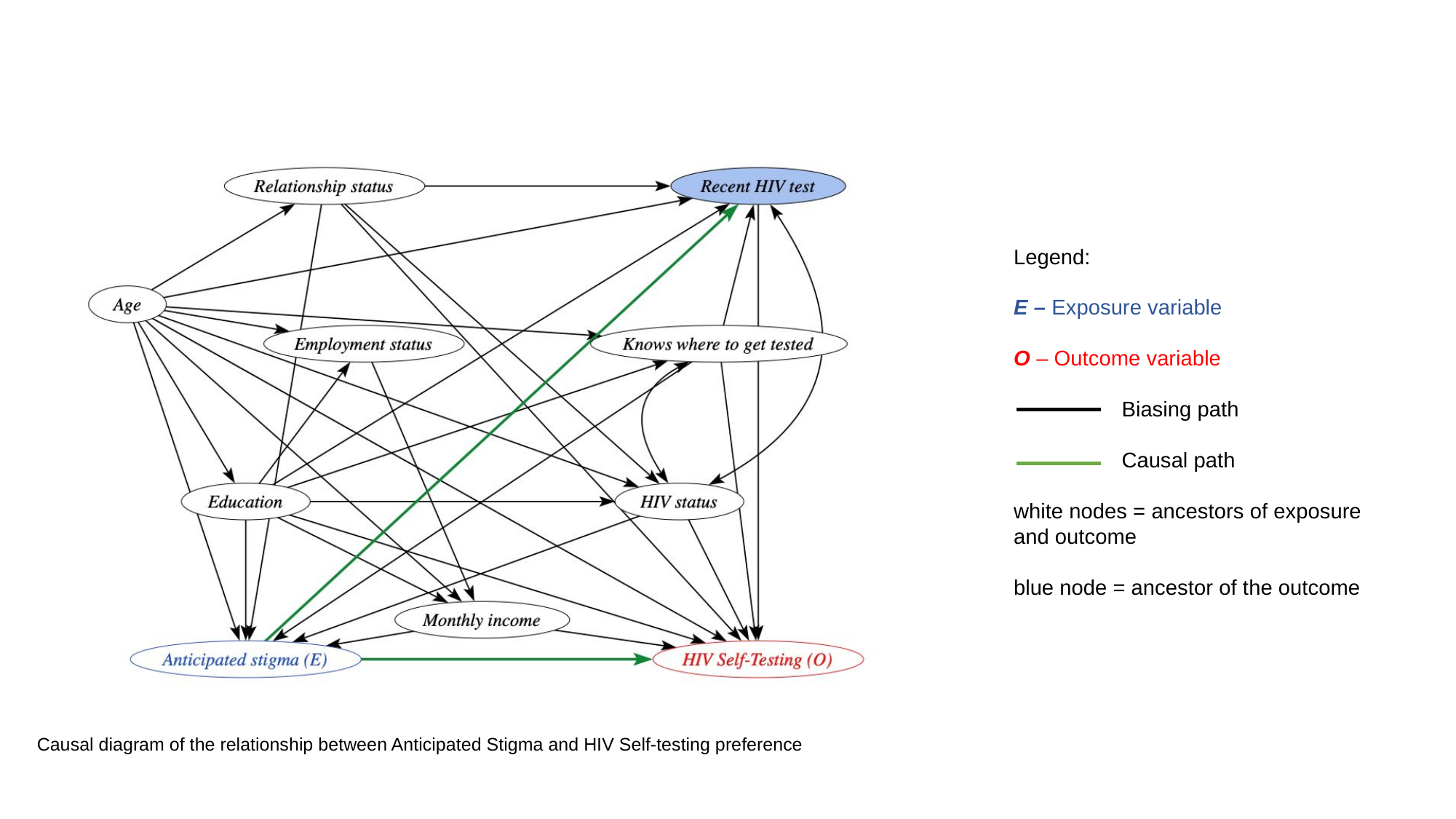

Legend:
E – Exposure variable
O – Outcome variable
 Biasing path
 Causal path
white nodes = ancestors of exposure and outcome
blue node = ancestor of the outcome
Causal diagram of the relationship between Anticipated Stigma and HIV Self-testing preference

## Slide 2
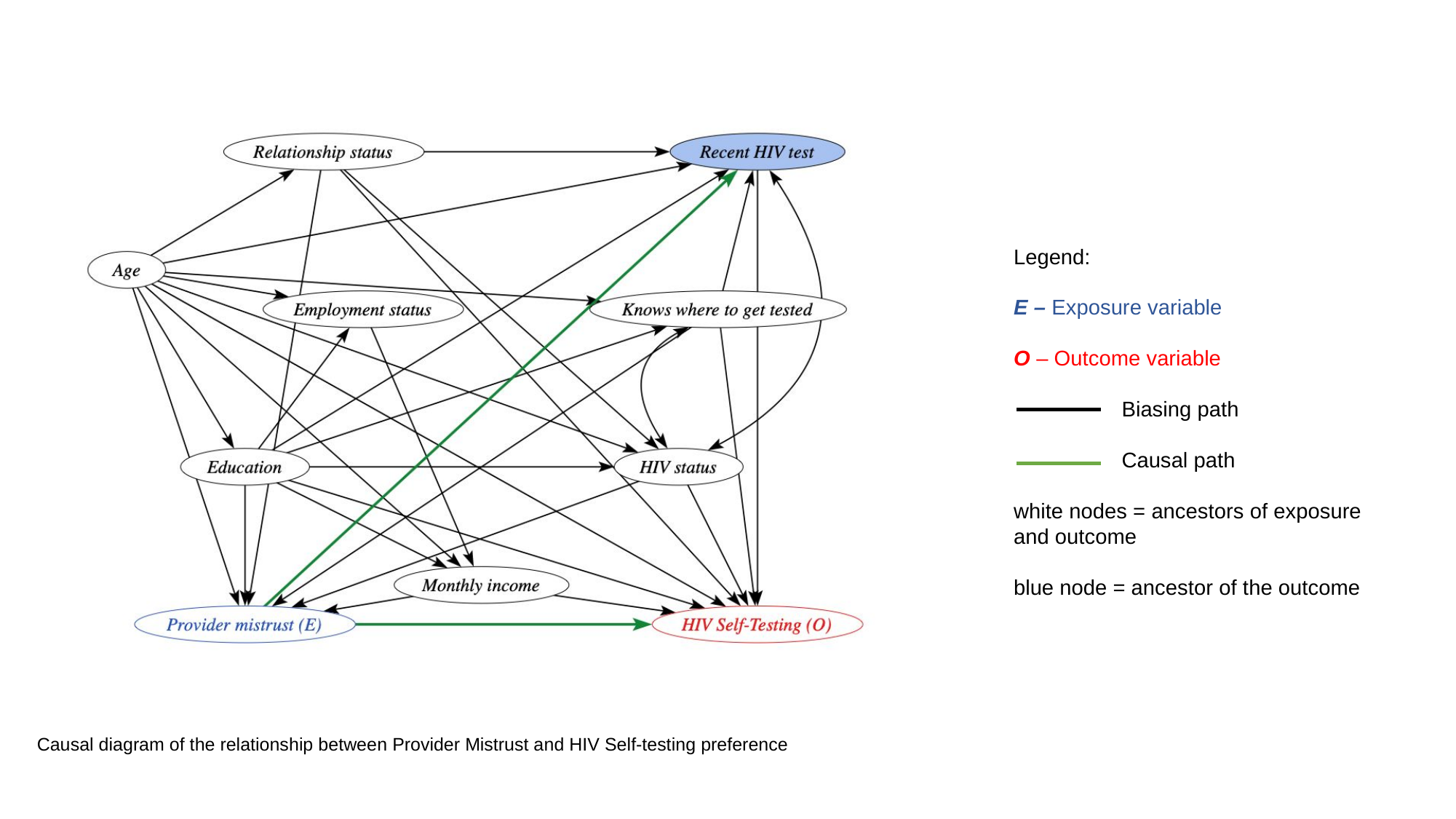

Legend:
E – Exposure variable
O – Outcome variable
 Biasing path
 Causal path
white nodes = ancestors of exposure and outcome
blue node = ancestor of the outcome
Causal diagram of the relationship between Provider Mistrust and HIV Self-testing preference

## Slide 3
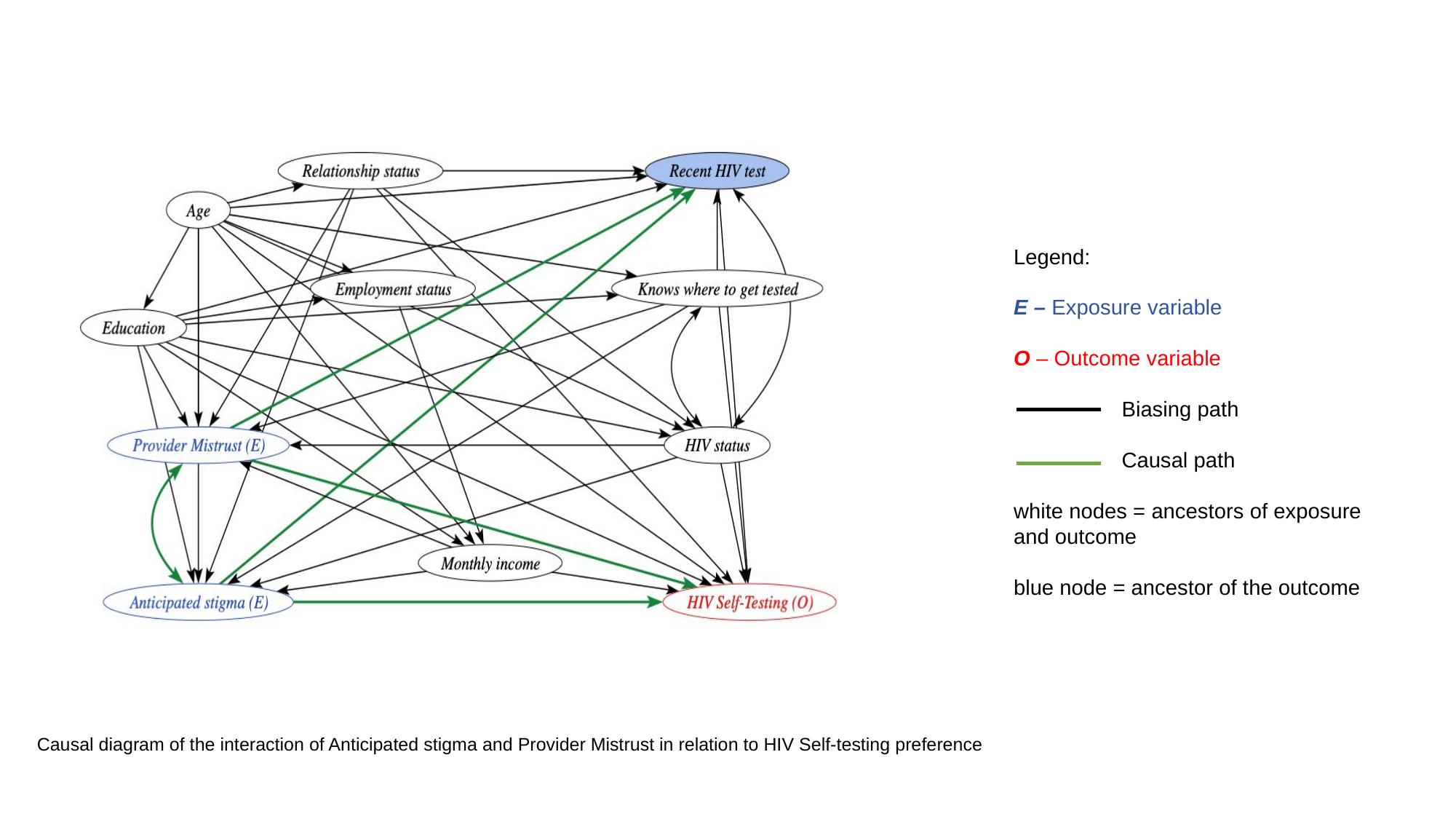

Legend:
E – Exposure variable
O – Outcome variable
 Biasing path
 Causal path
white nodes = ancestors of exposure and outcome
blue node = ancestor of the outcome
Causal diagram of the interaction of Anticipated stigma and Provider Mistrust in relation to HIV Self-testing preference
